# Usefulness scale for patient information material (USE) - development and psychometric properties

**DOI:** 10.1186/s12911-015-0153-7

**Published:** 2015-04-19

**Authors:** Lars P Hölzel, Zivile Ries, Jörg Dirmaier, Jördis M Zill, Levente Kriston, Christian Klesse, Martin Härter, Isaac Bermejo

**Affiliations:** Research Group on Psychotherapy and Health Services Research, Department of Psychiatry and Psychotherapy, Medical Center - University of Freiburg, Freiburg, Germany; Department of Medical Psychology, University Medical Center Hamburg-Eppendorf, Hamburg, Germany; Rhein-Jura-Klinik, Bad Säckingen, Germany; Medical Center - University of Freiburg, Freiburg, Germany

**Keywords:** Usefulness, Psychometrics, Patient empowerment, Medical education, Patient information, Pamphlet

## Abstract

**Background:**

One economical way to inform patients about their illness and medical procedures is to provide written health information material. So far, a generic and psychometrically sound scale to evaluate cognitive, emotional, and behavioral aspects of the subjectively experienced usefulness of patient information material from the patient’s perspective is lacking. The aim of our study was to develop and psychometrically test such a scale.

**Methods:**

The Usefulness Scale for Patient Information Material (USE) was developed using a multistep approach. Ultimately, three items for each subscale (cognitive, emotional, and behavioral) were selected under consideration of face validity, discrimination, difficulty, and item content.

The final version of the USE was subjected to reliability analysis. Structural validity was tested using confirmatory factor analysis, and convergent and divergent validity were tested using correlation analysis. The criterion validity of the USE was tested in an experimental design. To this aim, patients were randomly allocated to one of two groups. One group received a full version of an information brochure on depression or chronic low back pain depending on the respective primary diagnosis. Patients in the second group received a reduced version with a lower design quality, smaller font size and less information.

Patients were recruited in six hospitals in Germany. After reading the brochure, they were asked to fill in a questionnaire.

**Results:**

Analyzable data were obtained from 120 questionnaires. The confirmatory factor analysis supported the structural validity of the scale. Reliability analysis of the total scale and its subscales showed Cronbach’s α values between .84 and .94. Convergent and divergent validity were supported. Criterion validity was confirmed in the experimental condition. Significant differences between the groups receiving full and reduced information were found for the total score (p<.001) and its three subscales (cognitive p<.001, emotional p=.001, and behavioral p<.001), supporting criterion validity.

**Conclusions:**

We developed a generic scale to measure the subjective usefulness of written patient information material from a patient perspective. Our construct is defined in line with current theoretical models for the evaluation of written patient information material. The USE was shown to be a short, reliable and valid psychometric scale.

**Electronic supplementary material:**

The online version of this article (doi:10.1186/s12911-015-0153-7) contains supplementary material, which is available to authorized users.

## Background

Written patient information material (PIM) is often used in medical settings as an economical way to provide medical information to patients. A large body of guidelines and recommendations exists on how to develop high-quality information material [1] and how to evaluate its quality from an expert perspective [2-4]. So far, the focus of evaluation strategies has mainly been on whether the information material fulfills certain criteria regarding structural quality (e.g., completeness of content, information sources), readability, or comprehensibility. For example, the Suitability Assessment of Material (SAM) instrument measures the suitability of PIM from an expert point of view [5]. Nevertheless, it has been criticized that to date, existing approaches lack an explicit and theory-based evaluation model [6]. Therefore, we aimed to develop and psychometrically test a generic scale to evaluate PIM from a patient perspective under consideration of theoretical assumptions.

An analysis conducted by Dixon-Woods of publications about the use of printed PIM revealed two different theoretical approaches [7]. The first, which Dixon-Woods calls the “patient education” approach, is interested in the improvement of biomedical outcomes. According to this approach, patients are seen as passive information recipients, and a mechanistic model of communication (transmitter, receiver, effect) is applied. Information material in this model aims to educate patients about what is medically “correct” and tries to bring about cognitive, attitudinal, or behavioral changes [7]. The second approach is the “patient empowerment” approach, which aims to promote informed choice of patients. According to this aim, communication is seen as an active process of constructing the subjective meanings of a text [7]. Based on this second approach, Garner et al. proposed an evaluation model which they call the “*tripartite model of reading for evaluating and enhancing”* PIM [6]. In this model, making sense of PIM involves three processes: (1) reading the PIM, (2) constructing a coherent meaning of the PIM, and (3) responding to the content of the PIM [6]. The reader’s response to the material - including cognitive, affective and often intentional/behavioral aspects - is seen in this model as the “ultimate test” of PIM [6]. For evaluation, the model suggests qualitative methods focusing on compliance with the objective of the brochures and on the understanding of the PIM, which the authors term “communicative effectiveness”. The patient’s answers are judged by an interviewer and not by the patient him/herself. In line with this model, we aimed to measure cognitive, affective, and (intended) behavioral responses to PIM. However, in contrast to the proposal by Garner and colleagues, we wanted the patient to judge the benefit of using the PIM on his/her cognitions, emotions and (intended) behavior according to the notion of “patient empowerment”. In order to distinguish our self-reported judgment from “communicative effectiveness” as proposed by Garner et al., we named it “subjective usefulness”. PIM can be judged regarding subjective usefulness on three levels:

1) On the level of *cognitions*, PIM can improve patients’ knowledge; 2) on the level of *emotions*, PIM can help patients to cope with their illness; and 3) on the level of (intended) *behavior*, PIM can inform patients about what they can do to manage their illness. These effects may or may not be perceived as useful. Accordingly, we aimed to measure 1) the extent to which the patient perceives the knowledge gain as useful rather than the understanding of PIM or the objectively measurable gain in knowledge; 2) the perceived usefulness of the material in terms of coping with an illness; and 3) the extent to which the patient judges the behavior-related information to be useful to him/her, rather than compliance with the PIM’s suggestions.

In the following, we distinguish our construct of “subjective usefulness” from related constructs. As opposed to satisfaction, the construct of “subjective usefulness” implies a more active patient role. Usefulness implies the intended use of the information for understanding, gaining control, coping, or taking action. By contrast, it is possible for patients to be satisfied with information but nevertheless remain passive.

Furthermore, the construct of “subjective usefulness” has commonalities with the “Knowledge-Attitude-Behavior” (KAB) model [8], the “Technology-Acceptance Model” (TAM) [9,10] and the model of “Information Mastery” [11]. While the KAB aims to explain behavioral changes and the TAM seeks to explain technology use, our construct serves another purpose. PIM may have effects on a cognitive, emotional and behavioral level, and we aimed to explore whether patients perceive this effect as being of beneficial use to them. Another difference is that while the KAB and TAM describe a linear process, we see the relation between cognition, emotion and behavior as reciprocal. A further related construct is “Information Mastery”, in which the usefulness of information has been described with respect to clinicians in the form of an equation, considering the characteristics of relevance, validity and work. According to this equation, usefulness is high if the information has a high relevance and high validity, and little work is needed to receive the information [11]. While this equation aims to explain the relationship between specific characteristics of usefulness of information for clinicians, the construct of “subjective usefulness” is a post hoc judgment of PIM from a patient perspective.

In summary, the aim of our study was to develop and psychometrically test a generic scale measuring the “subjective usefulness” of PIM taking into account cognitive, emotional, and behavioral aspects.

## Methods

### Development

To develop and test the Usefulness Scale for Patient Information Material (USE), we applied a multistep approach.

First, based on the theoretical considerations outlined above, the authors formulated a *preliminary pool* of 48 new items to measure the cognitive, emotional, and behavioral dimension of usefulness of PIM.

Second, the *preliminary item pool* was rated regarding face validity (the extent to which an item appears to measure usefulness) and wording, including independent external raters (N = 18). All items were assessed on a scale from 1 (“very good”) to 5 (“insufficient”). The raters were also invited to suggest adaptations of wording using a free-text format. The allocation of the items to the corresponding dimensions (cognitive, emotional, and behavioral) was concealed. All raters were asked to assign each item to one of three dimensions. All raters were researchers and were experienced in test development and psychometrics. The suggested adaptations of the wording were discussed by the authors and items were changed if necessary. Items with insufficient face validity were excluded. Through this process, the pool was reduced to 37 items.

Third, the *reduced item* pool was tested in a sample of patients with depressive disorders or chronic low back pain (see Procedures). The reduced item pool was subjected to item analysis including the calculation of discrimination (corrected item-total correlation) and difficulty (mean) for each item.

Finally, three items for each subscale (cognitive, emotional, and behavioral subscale) were selected under consideration of face validity, discrimination, difficulty, and item content, with the aim of covering all relevant aspects of the construct. The *final item pool* of the USE consists of the nine items.

### Reliability

The final version of the USE was subjected to reliability analysis by quantifying internal consistency. Internal consistency measures whether different items of a scale produce similar scores. Cronbach’s α [12] (as a common measure for internal consistency) was calculated for the whole scale and additionally for each subscale. Discrimination (corrected item-total correlation) and difficulty (mean) were again computed for each item.

### Validation

The validation of the USE was investigated using the data employed for reliability analyses in the development process. Structural, convergent, and criterion validity were estimated. The structural (factorial) validity of the scale was tested using confirmatory factor analysis [9-13]. The convergent validity of the USE was tested by investigating the correlations between the USE and three general usefulness items (see Instruments). Finally, the criterion validity of the USE was tested using an experimental design. Inpatients were randomly assigned to one of two groups: participants of the first group (“full information group”) received a full version of an information brochure on depression or chronic low back pain depending on the respective primary diagnosis. These brochures were developed by the authors within the project “Culture-Sensitive Patient Information for Patients with a Migration Background and a Chronic Disease” [13] (registration number: German Clinical Trials Register DRKS00004241; Universal Trial Number U1111-1135-8043). Patients in the second group (“reduced information group”) received a reduced version with a lower design quality, smaller font size and less information.

For depression, the reduced version included a brief description of depression, including common symptoms. The full version provided additional information on prevalence, gender differences, and an anti-stigma message (“depression can affect anybody”) as well as information on the etiology of depression. Both versions included general information on the main treatment goals and treatment options (antidepressant medication/psychotherapeutic treatment). The full version included additional information on the options and a list of central preconditions, advantages and disadvantages of the treatments. A brief description of central health care providers was included in both brochures. Information on health care cost assumption and information on individual self-help was only provided in the full version. Illustrative examples and sources for additional information were given in both versions, while references were only provided in the full version.

For chronic low back pain, both brochures included general information on the disorder, including a description of symptoms. Additional information on prevalence and gender differences was included in the full version. Diagnostic measures were included in both brochures, while background information on diagnostic measures was only included in the full version of the brochure. Information on the etiology of chronic low back pain was included in the full version but not in the reduced version. General information on treatment options was provided in both versions, while in-depth information on the rationale of treatments (especially regarding exercises) and on health care providers was only included in the full version. Both brochures included illustrative examples and sources for additional information. References were only provided in the full version.

In accordance with the framework of Garner et al. [6], we assumed that the usefulness of the reduced version would be lower compared to the full version. Therefore, we hypothesized that as a result of our experimental intervention, usefulness would be rated higher in the “full information group” than in the “reduced information group”. To ensure the validity of the experimental condition, we compared both groups using an adapted version of a standardized and psychometrically tested instrument, the Consumer Information Rating Form (CIRF) [14].

### Procedures

Cross-sectional data were collected in a clinical sample of patients with depression (ICD-10: F32, F33 or F34.1) and patients with chronic low back pain (ICD-10: M54.5, M54.8, M54.9). Patients were excluded if they were unable to fill in a questionnaire due to cognitive impairment or insufficient language skills.

Patients were recruited by their responsible physician/therapist in six hospitals (Rhein-Jura Klinik, Celenus Klinik Ortenau, Celenus Klinik Sigmund-Weil, Celenus Klinik Teufelsbad, Deutsche Klinik für Intergrative Medizin, and Klinik und Poliklinik für Psychiatrie und Psychotherapie am Universitätsklinikum Hamburg-Eppendorf) between July 2012 and July 2013. Eligible patients were informed about the study. Those who gave informed consent were asked to read a brochure either on chronic low back pain or depression depending on their current diagnosis. After reading, they were asked to fill out a four-page self-assessment questionnaire. As psychoeducation on the indications was part of the inpatient treatment process, we could ensure that all patients received all relevant medical information till the end of the inpatient treatment. The study was carried out in accordance with the Code of Ethics of the Declaration of Helsinki and was approved by the Ethics Committees of the Universities of Freiburg (No: 213/12) and Hamburg (No: MC-309/12), Germany.

### Instruments

The four-page self-assessment questionnaire included the USE, three global items to measure usefulness, questions on demographic characteristics, previous knowledge about the respective illness, and the CIRF (adaptation of the scale is described below).

The three global items regarding usefulness were self-constructed and were designed to have a high face validity (“All in all, the brochure was useful to me.”, “I will recommend the brochure.”, “If needed, I’m going to read the brochure again”).

Self-reported demographic information (sex, age, level of education, migration status) and data on previous knowledge about the illness were additionally collected using self-constructed items.

The CIRF is a scale that was developed to measure consumers’ perception of the comprehensibility, utility, and design quality of written medicine information (i.e. package inserts) [14]. Some items could be easily transferred to evaluate PIM instead of package inserts. However, we had to change some aspects of the CIRF to suit our study: In the *consumer comprehensibility rating scale*, we decided to exclude one item (No. 5: “Overall, how easy or hard would you say this information sheet is to keep for future reference?”) because it had shown a very low factor loading in a previous study [14]. The *utility rating scale* and the *consumer design quality rating scale* had to be adapted as they were concerned with the content of the medicine information. The items were changed to fit the new subject. The *utility rating scale* was adapted to measure the amount of information and its utility for the specific content of the brochure that was used.

Questionnaires with more than 30% of all items missing, and questionnaires with stereotypical responses, e.g. without any variation for at least one page of the questionnaire even though items were reverse-scored, were excluded. In the case of missing item data (only scales), up to 30% were replaced using the expectation-maximization algorithm (EM).

All analyses were performed using IBM SPSS Statistics/AMOS 20.0.0.

## Results

### Development

The results regarding face validity, dimensionality, discrimination, and difficulty are displayed in Additional file 1: Table S1. According to the expert ratings, 31 of the 37 items had an appropriate face validity” (mean ≤ 2; i.e., at least “good” at average). Correct item allocation to the predefined conceptual dimension varied between 22% and 100%. The corrected item-total correlations (discrimination) ranged from 0.15 to 0.89. Twenty-five items exceeded a corrected item-total correlation of 0.7. Difficulty varied between 4.72 and 8.42 on a scale from 0-10.

Based on these findings, we excluded all items with a face validity rating of more than two on average, a dimensionality of less than 70% correct allocations, a discrimination of less than .7 and a difficulty of >7. From the remaining 16 items, we chose 3 for each subscale with the aim of covering all relevant aspects of the construct (see Additional file 2).

### Sample characteristics

We recruited 134 participants, 120 of whom (89.6%) provided analyzable data. About 1.4% of the total data points were imputed using EM. Demographic characteristics and level of previous knowledge are reported in Table 1. Participants had an average age of approximately 48 years, and slightly more women than men participated in the study. Twelve patients reported having a migration background. About half of the participants had a “high-track” level of education (Fachhochschulreife, Abitur). On average, patients reported having moderate to good previous knowledge about their respective illness. There were no significant differences with respect to sample characteristics between patients of the “full information group” and patients of the “reduced information group”.

**Table 1:** **Sample characteristics**

	**Full information group (n = 57)**	**Reduced information group (n = 63)**	**Total sample(n = 120)**	**p**
**Demographic characteristics**				
**Age; yrs.**				
Mean (SD)	47.1 (12.4)	49.3 (11.1)	48.3 (11.7)	.303
**Sex; n (%)**				
Female	38 (66.7)	35 (55.6)	73 (60.8)	.213
Male	19 (33.3)	28 (44.4)	47 (39.2)	
**Migration status; n (%)**				
Migrant	8 (14.5)	4 (6.5)	12 (10.3)	.150
Non-migrant	47 (85.5)	58 (93.5)	105 (89.7)	
**Level of education; n (%)**				
Low-track	6 (10.5)	11 (17.7)	17 (14.3)	.520
Middle-track	17 (29.8)	18 (29.0)	35 (29.4)	
High-track	34 (59.6)	33 (53.2)	67 (56.3)	
**Clinical characteristics**				
**Illness; n (%)**				
Depression	42 (73.7)	41 (65.1)	83 (69.2)	.308
Chronic low back pain	15 (26.3)	22 (34.9)	37 (30.8)	
**Previous knowledge**				
Mean (SD)	3.6 (0.9)	3.6 (1.0)	3.6 (1.0)	.966

n = number, mean = mean score, SD = standard deviation, Level of education: low-track schools [keinen Schulabschluss, Volksschul- oder Hauptschulabschluss], middle-track schools [Realschulabschluss/Mittlere Reife], high-track schools [Fachhochschulreife, Abitur].

### Reliability

Reliability analysis of the total scale showed a Cronbach’s α of .94 (see Table 2). The Cronbach’s α for the subscales were .84 for the cognitive, .94 for the emotional and .91 for the behavioral subscale. Corrected item-total correlations ranged from .67 to .86 for the total scale, from .69 to .71 for the cognitive, from .80 to .92 for the emotional and from .80 to .86 for the behavioral subscale.

**Table 2:** **Reliability analyses**

**Item**	**Discrimination (corrected item-total correlation)**	**α of the total scale**	**Subscales**	**Discrimination (corrected item-subscale correlation)**	**α of the subscales**
1	0.75	0.94	Cognitive	0.71	0.84
2	0.69			0.69	
3	0.67			0.71	
4	0.67		Emotional	0.80	0.94
5	0.83			0.92	
6	0.86			0.89	
7	0.86		Behavioral	0.82	0.91
8	0.83			0.86	
9	0.75			0.80	

*Note.* α refers to Cronbach’s α; mean = mean score; SD = standard deviation.

### Validity

**Figure 1:**
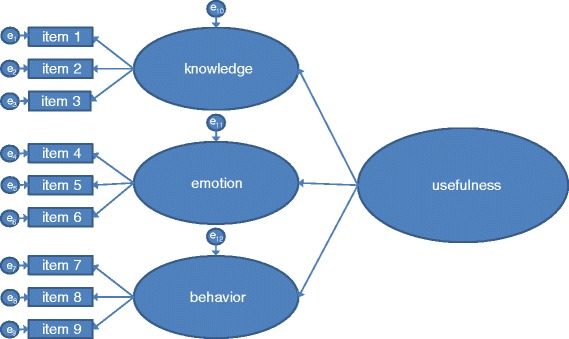
Structure of the theoretical model.

The structure of our theoretical model (see Figure 1) was largely confirmed by a confirmatory factor analysis (CFA).

Local goodness-of-fit indices suggested a good fit between the theoretical model and the data (see Table 3). Factor loadings of all items, as well as construct reliability and average variance extracted per subscale, reached common thresholds. Factor loadings of the subscales on the second-order factor (usefulness) were .90 for knowledge, .83 for emotion and .97 for behavior. The construct reliability was .93 and the extracted variance was .82. Results regarding global goodness-of-fit indices were more heterogeneous. The Tucker-Lewis Index (TLI) and the Comparative Fit Index (CFI) indicated a good fit to the data (TLI = .96, CFI = .97), and the chi-square to degrees of freedom ratio spoke in favor of the model (χ^2^/df = 2.14). However, the Root Mean Square Error of Approximation indicated that there is still about 10% of variance unexplained in the model (RMSEA = .10) [7-11].

**Table 3:** **Local goodness-of-fit indices**

**Subscale**	**Item**	**Factor loading**	**Construct reliability**	**Variance extracted**
	***Threshold for acceptable fit***	**≥** ***0.4***	**≥** ***0.6***	**≥** ***0.5***
Cognitive	1	0.82	0.84	0.64
	2	0.78		
	3	0.79		
Emotional	4	0.82	0.94	0.83
	5	0.97		
	6	0.96		
Behavioral	7	0.91	0.91	0.78
	8	0.91		
	9	0.83		

*Note.* Recommendations are based on [16-20].

The *convergent validity* of the USE was supported by substantial correlations between the USE scores (total score and subscales) and the three general items (see Table 4).

**Table 4:** **Convergent validity**

	**“All in all, the brochure was useful to me.”**	**“I will recommend the brochure.”**	**“If needed, I’m going to read the brochure again.”**
**Total scale**	.68**	.61**	.62**
**Subscales**			
Cognitive	.72**	.61**	.67**
Emotional	.57**	.50**	.55**
Behavioral	.57**	.54**	.47**

*Note.* **statistically significant Pearson product-moment correlations (p < .001).

The *discriminant validity* of the three subscales was tested by investigating the inter-correlation of the three subscales. The correlation between the cognitive and the emotional subscale was .64 (p < .001), between the cognitive and behavioral subscale .76 (p < .001) and between the emotional and behavioral subscale .72 (p < .001). Accordingly, the shared variance between the subscales was between 41% and 58%.

The integrity of the intervention was investigated by using the adapted version of the CIRF. A significant effect of the content of the brochures was found in two of three subscales of the adapted version of the CIRF. While a mean score of 22.77 was found for the utility subscale of the full version, the reduced version reached a significantly (p < .001) lower score of 19.21 (theoretical range 7-28, with 7 indicating low utility and 28 indicating high utility). The comprehensiveness of the full version of the brochure was rated as slightly higher (16.77) than the comprehensiveness of the reduced version (16.13). This difference was not statistically significant (p = .215). Design quality rating was again found to be significantly (p < .001) higher for the full version (33.39) than for the reduced version (28.52).

The criterion validity of the USE was confirmed by comparing ratings of the two versions. The total score of the USE was approximately 20 points higher in the group with the full version of the PIM than in the group with the reduced material (see Table 5). This difference was statistically significant (p < .001). Significant differences between the two groups were found for all subscales.

**Table 5:** **Criterion validity**

	**EG (n = 57)Mean (SD)**	**CG (n = 63)Mean (SD)**	**p**
**Total score** ^**1**^	62.82 (17.49)	44.17 (20.62)	<.001
**Subscales** ^**2**^			
Cognitive	22.05 (5.84)	15.94 (7.46)	<.001
Emotional	18.61 (7.65)	13.90 (8.90)	.001
Behavioral	22.16 (6.57)	14.33 (7.65)	<.001

*Note.* EG = experimental group; CG = control group; n = number; mean = mean score; SD = standard deviation; 1 = range 0-90; 2 = range 0-30.

## Discussion

In this study, we developed a scale to measure the subjectively experienced usefulness of PIM from a patient perspective. The total score of the final version is a generic estimate, while the three subscales can be used to differentiate between cognitive, emotional, and behavioral aspects of subjective usefulness. The USE turned out to be a reliable and valid scale.

The theoretical model with three factors (subscales) and one second-order factor (total score) was investigated using a confirmatory factor analysis. Although a number of indices indicated a good fit between the model and the data, approximately 10% of the variance could not be explained by the model. As we collected data in several hospitals, with two different indications, and employed an experimental intervention, and given that there were large differences in previous knowledge, this may have caused additional variance. Due to the comparatively small sample size, we were unable to include these variables in our model. Moreover, our items may share some aspects (such as wording) which are not taken into account by the model. Although the percentage of unexplained variance in the model is about 2% above common thresholds, we conclude that the structural validity of the scale was largely supported by the confirmatory factor analysis.

Although the USE is a very short scale with only 9 items (3 items per subscale), it proved to be reliable. The internal consistency (Cronbach’s α) of the scale and its subscales was high. Factor reliability values investigated in the confirmatory factor analysis were in line with the results of the internal consistency.

Convergent validity was confirmed, as the total score of the USE and all subscales showed substantial correlations with a general statement about the usefulness of the brochure, a statement about whether the participants would recommend the brochure to others, and a statement about whether they would read it again. Pluye et al. have recently published a questionnaire to evaluate online health information for patients and health information consumers, the Information Assessment Method (IAM) [15]. This questionnaire is a candidate for investigating the concurrent validity of the USE in future studies. The discriminant validity between the three subscales was investigated. As expected, due to the presence of a common second-order factor, significant moderate to high correlations between the three subscales were found. The association is sufficiently high to argue that the three subscales are part of the same construct, and the fact that there is still unique variance left in every scale is in line with our model of three closely related but not redundant constructs (see Figure 1). Support for criterion validity was also found, as the scales were able to distinguish between groups of patients who received information material of low vs. high usefulness. The integrity of the intervention, i.e., a true difference between the two groups receiving information materials of different quality, was also confirmed by additional instruments.

To enable the utilization of the USE beyond German-speaking countries, the items were translated from German into English, Italian, Polish, Russian, and Turkish by two independent professional translators (see Additional files 3, 4, 5, 6, 7). A consensus version was developed by a third professional translator and back-translated into German by a fourth professional translator. The original wording was compared to the back-translated version by the translator, who developed the consensus version together with the authors to confirm the accuracy of the translation process.

Although the psychometric soundness of the scale was confirmed in our study, some limitations remain. First, as our sample size was small for using confirmatory factor analysis, we developed and tested the model in the same sample. A replication of our results in an independent sample is therefore required. Second, although we provide the USE in English, German, Italian, Polish, Russian, and Turkish, the psychometric properties of the scales were evaluated exclusively in a German-speaking sample. Third, even though the items of the scale are designed to measure the usefulness of PIM in a generic manner, our scale was investigated exclusively in inpatients suffering from depression or chronic low back pain. A cross-validation of our findings using other language versions of the USE, different treatment settings, and different indications is therefore desired. The investigation of the convergent validity of the scale was limited, as we had to use generic items which themselves have not been previously validated. This was necessary because items and scales to measure the usefulness of PIM are largely lacking to date. There is a substantial overlap between the three subscales of the USE and the Knowledge-Attitude-Behavior (KAB) Model [8], the Technology-Acceptance Model (TAM) [9,10] and Information Mastery [11]. Investigations into the relationships between these constructs would be an interesting subject for further investigations. Although further studies on construct validity are required, we found evidence on criterion validity. By systematically varying the content of the brochures, we were able to experimentally manipulate the usefulness of PIM. We are convinced that this experimental design has a high internal and external validity and provides a strong criterion for an instrument which aims to measure the usefulness of PIM. Qualitative investigations into patients’ expectations about PIM and views regarding what hinders or facilitates usefulness may provide further starting points for investigating the criterion validity of the USE.

## Conclusion

Based on current theoretical evaluation models, we developed a generic scale (USE) to measure the subjectively experienced usefulness of PIM from a patient perspective. The USE proved to be a short, reliable and valid psychometric scale.

## Additional files

Additional file 1: Table S1. Characteristics of the preliminary item pool. Additional file 2:
**Usefulness scale for patient information material (USE) – German.**
Additional file 3:
**Usefulness scale for patient information material (USE) – English.**
Additional file 4:
**Usefulness scale for patient information material (USE) – Italian.**
Additional file 5:
**Usefulness scale for patient information material (USE) – Polish.**
Additional file 6:
**Usefulness scale for patient information material (USE) – Russian.**
Additional file 7:
**Usefulness scale for patient information material (USE) – Turkish.**

## Abbreviations

USE: Usefulness Scale for Patient Information Material
PIM: Patient information material
KAB: Knowledge-Attitude-Behavior Model
TAM: Technology-Acceptance Model
CIRF: Consumer Information Rating Form
EM: Expectation-maximization algorithm
CFA: Confirmatory factor analysis
TLI: Tucker-Lewis Index
CFI: Comparative Fit Index
RMSEA: Root Mean Square Error of Approximation
