# Supplementary material for: Usefulness scale for patient information material (USE) - development and psychometric properties
Source: BMC Med Inform Decis Mak. 2015 Apr 19;15:34. doi: 10.1186/s12911-015-0153-7 (PMC4456699; doi:10.1186/s12911-015-0153-7)
Supplement: Additional file 1: Table S1. — Characteristics of the preliminary item pool. [file 12911_2015_153_MOESM1_ESM.pdf]

Supplementary Table 1: Characteristics of the preliminary item pool

**Subscale**	**Item**	**Face validity (mean; range 1-5*)**	**Dimensionality (correct allocation in %)**	**Discrimination (corrected item-total correlation)**	**Difficulty (mean; range 0-10)**
Knowledge	a	2.5	86.7	.83	6.78
	b	1.7	100.0	.83	6.52
	c	2.0	100.0	.86	6.11
	d	2.0	100.0	.87	6.19
	**e**	**1.8**	**86.7**	**.84**	**6.03**
	f	1.6	100.0	.55	7.41
	g	1.7	79.0	.81	6.35
	h	1.5	100.0	.69	7.70
	i	2.0	100.0	.64	4.72
	j	1.5	100.0	.78	6.08
	k	1.8	88.2	.71	5.10
	**l**	**1.6**	**87.5**	**.77**	**6.04**
	m	1.2	33.3	.63	7.62

	n	1.6	66.7	.60	7.78
	o	2.1	93.8	.72	7.38
	**p**	**1.8**	**93.8**	**.76**	**6.78**
Emotion	a	2.3	22.2	.57	6.79
	b	2.0	80.0	.76	5.55
	c	1.7	25.0	.83	4.90
	d	2.3	23.5	.76	5.28
	e	1.8	100.0	.15	8.42
	f	1.5	93.8	.62	4.72
	**g**	**1.6**	**100.0**	**.74**	**4.81**
	**h**	**1.7**	**100.0**	**.89**	**5.55**
	**i**	**1.6**	**100.0**	**.87**	**5.78**
	j	1.2	100.0	.21	8.17
	k	1.7	36.8	.81	5.42
Behavior	a	1.6	81.5	.81	5.98
	b	1.7	26.7	.63	5.81
	**c**	**1.8**	**75.0**	**.88**	**5.78**
	d	1.3	81.3	.85	6.00

**e**	**1.2**	**75.0**	**.85**	**5.74**
f	1.8	66.7	.80	6.26
g	1.5	77.8	.78	6.39
**h**	**1.3**	**73.7**	**.86**	**6.53**
i	2.2	66.7	.58	6.70
j	2.3	82.4	.36	5.49

Note. *range 1=”very good” to 5=”insufficient”; mean=mean score; bold type=items selected
