# Supplementary material for: Usefulness scale for patient information material (USE) - development and psychometric properties
Source: BMC Med Inform Decis Mak. 2015 Apr 19;15:34. doi: 10.1186/s12911-015-0153-7 (PMC4456699; doi:10.1186/s12911-015-0153-7)
Supplement: Additional file 2: — Usefulness scale for patient information material (USE) – German. [file 12911_2015_153_MOESM2_ESM.doc]

| Bewerten Sie bitte im Folgenden jede Aussage, indem Sie angeben, inwieweit Sie der Aussage zustimmen. Wenn Sie einer Aussage überhaupt nicht zustimmen, kreuzen Sie bitte den Kreis ganz links an. Wenn sie der Aussage voll und ganz zustimmen, kreuzen Sie den Kreis ganz rechts an. Die Kreise dazwischen bieten Ihnen die Möglichkeit die stärke Ihrer Zustimmung auszudrücken.  Wenn Sie sich verschrieben haben, können Sie das Kreuz durchstreichen und neu ankreuzen. Bitte setzen Sie nur ein Kreuz pro Aussage.  *Ein Beispiel verdeutlicht, wie es geht:* Wenn die Broschüre Ihnen überhaupt nicht geholfen hat, die Behandlung der Erkrankung zu verstehen, setzen Sie an folgender stelle ein Kreuz:   | **Die Broschüre…** | |  | | | | --- | --- | --- | --- | --- | |  | *stimme*  *überhaupt*  *nicht zu*  X  *neutral*  X  *stimme*  *voll und*  *ganz zu* | |  |  | | …hat mir geholfen, die Behandlung zu  verstehen. | O····O····O····O····O····O····O····O····O····O····O | | | |   Bitte beantworten Sie jede Frage möglichst offen und ehrlich, so wie es für Sie persönlich zutrifft.  **Bitte bewerten Sie die folgenden Aussagen:**   |  | **Die Broschüre…** | | |  | | |  | | | --- | --- | --- | --- | --- | --- | --- | --- | --- | |  | *stimme*  *überhaupt*  *nicht zu* | *neutral* | *stimme*  *voll und*  *ganz zu* | |  | | 1. | | …enthält Informationen, die ich brauche. | O····O····O····O····O····O····O····O····O····O····O | | | | | | | 2. | | …hat mir geholfen, die Erkrankung zu verstehen. | O····O····O····O····O····O····O····O····O····O····O | | | | | | | 3. | | …hat mir geholfen, die Behandlungs-möglichkeiten zu verstehen. | O····O····O····O····O····O····O····O····O····O····O | | | | | | | 4. | | …hat meine Sorgen wegen meiner Erkrankung verringert. | O····O····O····O····O····O····O····O····O····O····O | | | | | | | 5. | | …hat mir Mut gemacht. | O····O····O····O····O····O····O····O····O····O····O | | | | | | | 6. | | …hat mir Hoffnung gegeben, dass ich mich wieder besser fühlen kann. | O····O····O····O····O····O····O····O····O····O····O | | | | | | | 7. | | …hilft mir mich an Entscheidungen über die Behandlung zu beteiligen. | O····O····O····O····O····O····O····O····O····O····O | | | | | | | 8. | | …hat mir gezeigt, wie ich selbst zum Erfolg der Behandlung beitragen kann. | O····O····O····O····O····O····O····O····O····O····O | | | | | | | 9. | | …hat mich ermutigt, selbst aktiv zu sein, damit mein Zustand sich verbessert. | O····O····O····O····O····O····O····O····O····O····O | | | | | | |
| --- | --- | --- | --- | --- | --- | --- | --- | --- | --- | --- | --- | --- | --- | --- | --- | --- | --- | --- | --- | --- | --- | --- | --- | --- | --- | --- | --- | --- | --- | --- | --- | --- | --- | --- | --- | --- | --- | --- | --- | --- | --- | --- | --- | --- | --- | --- | --- | --- | --- | --- | --- | --- | --- | --- | --- | --- | --- | --- | --- | --- | --- | --- | --- | --- | --- | --- | --- | --- | --- | --- | --- | --- | --- | --- | --- | --- | --- | --- | --- | --- | --- | --- | --- | --- | --- | --- | --- | --- | --- | --- | --- | --- | --- | --- | --- | --- | --- | --- | --- | --- | --- | --- | --- | --- | --- | --- | --- | --- | --- | --- | --- |
