# Supplementary material for: Usefulness scale for patient information material (USE) - development and psychometric properties
Source: BMC Med Inform Decis Mak. 2015 Apr 19;15:34. doi: 10.1186/s12911-015-0153-7 (PMC4456699; doi:10.1186/s12911-015-0153-7)
Supplement: Additional file 3: — Usefulness scale for patient information material (USE) – English. [file 12911_2015_153_MOESM3_ESM.doc]

| Rate each of the statements below to show the extent to which you agree with that statement. If you completely disagree with a statement, put a cross in the circle on the far left. If you completely agree with a statement, put a cross in the circle on the far right. Use the circles in between to express your level of agreement.  If you make a mistake, draw a line through the cross and put another in the right place. Please use only one cross per statement.  *This example shows how to do it:* If the brochure did not help you to understand the treatment of the disease/illness at all, place your cross as shown:   | **The brochure** | |  | | | | --- | --- | --- | --- | --- | |  | *completely*  *disagree*  X  *neutral*  X | | *completely*  *agree* |  | | …helped me to understand the treatment. | O····O····O····O····O····O····O····O····O····O····O | | | |     Please answer each question as openly and honestly as you can to indicate the way it applies to you  personally.  **Please rate each of the statements below:**   |  | **The brochure…** | | |  | | |  | | | --- | --- | --- | --- | --- | --- | --- | --- | --- | |  | *completely*  *disagree* | *neutral* | *completely*  *agree* | |  | | 1. | | …contains information I need. | O····O····O····O····O····O····O····O····O····O····O | | | | | | | 2. | | …helped me to understand the disease/illness. | O····O····O····O····O····O····O····O····O····O····O | | | | | | | 3. | | …helped me to understand the treatment options. | O····O····O····O····O····O····O····O····O····O····O | | | | | | | 4. | | …reduced my worries about my disease/illness. | O····O····O····O····O····O····O····O····O····O····O | | | | | | | 5. | | …has given me courage. | O····O····O····O····O····O····O····O····O····O····O | | | | | | | 6. | | …has given me the hope that I will feel better again. | O····O····O····O····O····O····O····O····O····O····O | | | | | | | 7. | | …helps me to participate in decisions made about my treatment. | O····O····O····O····O····O····O····O····O····O····O | | | | | | | 8. | | …showed me how I can contribute to the success of the treatment. | O····O····O····O····O····O····O····O····O····O····O | | | | | | | 9. | | …encouraged me to become more active in order to improve my condition. | O····O····O····O····O····O····O····O····O····O····O | | | | | | |
| --- | --- | --- | --- | --- | --- | --- | --- | --- | --- | --- | --- | --- | --- | --- | --- | --- | --- | --- | --- | --- | --- | --- | --- | --- | --- | --- | --- | --- | --- | --- | --- | --- | --- | --- | --- | --- | --- | --- | --- | --- | --- | --- | --- | --- | --- | --- | --- | --- | --- | --- | --- | --- | --- | --- | --- | --- | --- | --- | --- | --- | --- | --- | --- | --- | --- | --- | --- | --- | --- | --- | --- | --- | --- | --- | --- | --- | --- | --- | --- | --- | --- | --- | --- | --- | --- | --- | --- | --- | --- | --- | --- | --- | --- | --- | --- | --- | --- | --- | --- | --- | --- | --- | --- | --- | --- | --- | --- | --- | --- | --- | --- |
