# Supplementary material for: Usefulness scale for patient information material (USE) - development and psychometric properties
Source: BMC Med Inform Decis Mak. 2015 Apr 19;15:34. doi: 10.1186/s12911-015-0153-7 (PMC4456699; doi:10.1186/s12911-015-0153-7)
Supplement: Additional file 4: — Usefulness scale for patient information material (USE) – Italian. [file 12911_2015_153_MOESM4_ESM.doc]

| Valuti ciascuna delle seguenti affermazioni, indicando in che misura concorda con esse. Se non è affatto d'accordo con un'affermazione, apponga una crocetta sul primo cerchio a sinistra. Se è pienamente d'accordo con l'affermazione, apponga una crocetta sull'ultimo cerchio a destra. I cerchi intermedi servono per poter esprimere un giudizio intermedio.  Se avesse contrassegnato il cerchio sbagliato, può cancellare e apporre una nuova crocetta. La preghiamo di apporre una sola crocetta per ciascuna affermazione.  *Ecco un esempio chiarificatore:* se la brochure non l'ha aiutata affatto a comprendere in cosa consista  la terapia per la malattia, apponga una crocetta in questo punto:   | **La brochure...** | |  | | | | --- | --- | --- | --- | --- | |  | *non sono*  *affatto*  *d‘accordo*  X  *neutrale*  X  *sono*  *pienamente*  *d’accordo* | |  |  | | …mi ha aiutato a comprendere la terapia. | O····O····O····O····O····O····O····O····O····O····O | | | |   Risponda a ciascuna domanda il più apertamente e sinceramente possibile, in base alla Sua situazione  personale.  **Esprima un giudizio sulle seguenti affermazioni:**   |  | **La brochure...** | | |  | | |  | | | --- | --- | --- | --- | --- | --- | --- | --- | --- | |  | *non sono*  *affatto*  *d‘accordo* | *neutrale* | *sono*  *pienamente*  *d’accordo* | |  | | 1. | | ...contiene le informazioni di cui ho bisogno. | O····O····O····O····O····O····O····O····O····O····O | | | | | | | 2. | | ...mi ha aiutato a comprendere la malattia. | O····O····O····O····O····O····O····O····O····O····O | | | | | | | 3. | | ...mi ha aiutato a comprendere le possibilità di terapia. | O····O····O····O····O····O····O····O····O····O····O | | | | | | | 4. | | ...ha diminuito le mie preoccupazioni riguardo alla malattia. | O····O····O····O····O····O····O····O····O····O····O | | | | | | | 5. | | ...mi ha dato coraggio. | O····O····O····O····O····O····O····O····O····O····O | | | | | | | 6. | | ...mi ha dato la speranza di poter tornare a stare meglio. | O····O····O····O····O····O····O····O····O····O····O | | | | | | | 7. | | ...mi aiuta a partecipare alle decisioni relative alla terapia. | O····O····O····O····O····O····O····O····O····O····O | | | | | | | 8. | | ...mi ha mostrato come posso contribuire personalmente al successo della terapia. | O····O····O····O····O····O····O····O····O····O····O | | | | | | | 9. | | ...mi ha incoraggiato ad essere attivo/a al fine di migliorare le mie condizioni. | O····O····O····O····O····O····O····O····O····O····O | | | | | | |
| --- | --- | --- | --- | --- | --- | --- | --- | --- | --- | --- | --- | --- | --- | --- | --- | --- | --- | --- | --- | --- | --- | --- | --- | --- | --- | --- | --- | --- | --- | --- | --- | --- | --- | --- | --- | --- | --- | --- | --- | --- | --- | --- | --- | --- | --- | --- | --- | --- | --- | --- | --- | --- | --- | --- | --- | --- | --- | --- | --- | --- | --- | --- | --- | --- | --- | --- | --- | --- | --- | --- | --- | --- | --- | --- | --- | --- | --- | --- | --- | --- | --- | --- | --- | --- | --- | --- | --- | --- | --- | --- | --- | --- | --- | --- | --- | --- | --- | --- | --- | --- | --- | --- | --- | --- | --- | --- | --- | --- | --- | --- | --- |
