# Supplementary material for: Usefulness scale for patient information material (USE) - development and psychometric properties
Source: BMC Med Inform Decis Mak. 2015 Apr 19;15:34. doi: 10.1186/s12911-015-0153-7 (PMC4456699; doi:10.1186/s12911-015-0153-7)
Supplement: Additional file 5: — Usefulness scale for patient information material (USE) – Polish. [file 12911_2015_153_MOESM5_ESM.doc]

**Additional File 5: Usefulness scale for patient information material (USE) – Polish**

| Proszę ocenić poniżej każdą wypowiedź określając, w jakim stopniu zgadza się Pani/Pan z nią. Jeżeli w ogóle nie zgadza się Pani/Pan z daną wypowiedzią, proszę zaznaczyć pierwsze pole z lewej strony. Jeżeli w pełni zgadza się Pani/Pan z wypowiedzią, proszę o zaznaczenie pierwszego pola po prawej stronie. Pozostałe pola położone pomiędzy umożliwiają określenie, w jakim stopniu zgadzają się Państwo z daną wypowiedzią.  W przypadku popełnienia błędu może Pan/Pani przekreślić krzyżyk i zakreślić ponownie właściwe pole. Proszę postawić tylko jeden krzyżyk przy każdej wypowiedzi.  *Poniższy przykład ilustruje, jak to działa:* Jeżeli broszura wcale nie pomogła Pani/Panu zrozumieć leczenia choroby, proszę postawić krzyżyk w następującym polu:   | **Broszura…** | |  | | | | --- | --- | --- | --- | --- | |  | *całkowicie się*  *nie zgadzam*  X  *cena*  *neutralna*  X  *całkowicie*  *się zgadzam* | |  |  | | ...pomogła mi zrozumieć leczenie. | O····O····O····O····O····O····O····O····O····O····O | | | |   *Proszę odpowiedzieć na każde pytanie możliwie otwarcie i szczerze, zgodnie z własnymi odczuciami.*  **Proszę ocenić następujące stwierdzenia:**   |  | **Broszura…** | | |  | | |  | | | --- | --- | --- | --- | --- | --- | --- | --- | --- | |  | *całkowicie się*  *nie zgadzam* | *t cena*  *neutralna* | *całkowicie*  *się zgadzam* | |  | | 1. | | ...zawiera informacje, których potrzebuję. | O····O····O····O····O····O····O····O····O····O····O | | | | | | | 2. | | ...pomogła mi zrozumieć chorobę. | O····O····O····O····O····O····O····O····O····O····O | | | | | | | 3. | | ...pomogła mi zrozumieć dostępne możliwości leczenia. | O····O····O····O····O····O····O····O····O····O····O | | | | | | | 4. | | ...zmniejszyła moje obawy spowodowane chorobą. | O····O····O····O····O····O····O····O····O····O····O | | | | | | | 5. | | ...podniosła mnie na duchu. | O····O····O····O····O····O····O····O····O····O····O | | | | | | | 6. | | ...dała mi nadzieję, że znowu mogę poczuć się lepiej. | O····O····O····O····O····O····O····O····O····O····O | | | | | | | 7. | | ...zachęca mnie do uczestniczenia w podejmowaniu decyzji dotyczących leczenia. | O····O····O····O····O····O····O····O····O····O····O | | | | | | | 8. | | ...pokazała mi, w jaki sposób sam/a mogę przyczynić się do powodzenia leczenia. | O····O····O····O····O····O····O····O····O····O····O | | | | | | | 9. | | ...zachęciła mnie do bycia aktywnym, aby mój stan poprawił się. | O····O····O····O····O····O····O····O····O····O····O | | | | | | |
| --- | --- | --- | --- | --- | --- | --- | --- | --- | --- | --- | --- | --- | --- | --- | --- | --- | --- | --- | --- | --- | --- | --- | --- | --- | --- | --- | --- | --- | --- | --- | --- | --- | --- | --- | --- | --- | --- | --- | --- | --- | --- | --- | --- | --- | --- | --- | --- | --- | --- | --- | --- | --- | --- | --- | --- | --- | --- | --- | --- | --- | --- | --- | --- | --- | --- | --- | --- | --- | --- | --- | --- | --- | --- | --- | --- | --- | --- | --- | --- | --- | --- | --- | --- | --- | --- | --- | --- | --- | --- | --- | --- | --- | --- | --- | --- | --- | --- | --- | --- | --- | --- | --- | --- | --- | --- | --- | --- | --- | --- | --- | --- |
