# Supplementary material for: Usefulness scale for patient information material (USE) - development and psychometric properties
Source: BMC Med Inform Decis Mak. 2015 Apr 19;15:34. doi: 10.1186/s12911-015-0153-7 (PMC4456699; doi:10.1186/s12911-015-0153-7)
Supplement: Additional file 6: — Usefulness scale for patient information material (USE) – Russian. [file 12911_2015_153_MOESM6_ESM.doc]

| цените все приведенные далее высказывания, указав, насколько вы с ними согласны. Если вы совершенно не согласны с высказыванием, поставьте крестик в первом кружке слева. Если вы полностью согласны с высказыванием, поставьте крестик в первом кружке справа. Промежу-точные кружки позволят выразить степень вашего согласия с высказыванием.  Если вы поставили крестик не в том месте, вы можете его зачеркнуть и поставить новый крестик. Просьба не ставить больше одного крестика на высказывание.  *Обратите внимание на следующий пример:* если брошюра вам совсем не помогла понять процесс лечения заболевания, поставьте крестик в следующем месте:   | **Брошюра...** | |  | | | | --- | --- | --- | --- | --- | |  | совершенно не согласен(а)  X  нейтральное  отношение  X  полностью  согласен(а) | |  |  | | …помогла мне понять процесс лечения. | O····O····O····O····O····O····O····O····O····O····O | | | |   Просьба отвечать на каждый вопрос максимально честно, насколько высказывание верно  лично для вас.  **Пожалуйста, оцените следующие высказывания:**   |  | **Брошюра...** | | |  | | |  | | | --- | --- | --- | --- | --- | --- | --- | --- | --- | |  | совершенно не согласен(а) | нейтральное  отношение | полностью  согласен(а) | |  | | 1. | | …содержит необходимую мне информацию. | O····O····O····O····O····O····O····O····O····O····O | | | | | | | 2. | | …помогла мне понять суть заболевания. | O····O····O····O····O····O····O····O····O····O····O | | | | | | | 3. | | …помогла мне понять существующие возможности лечения. | O····O····O····O····O····O····O····O····O····O····O | | | | | | | 4. | | …уменьшила мое беспокойство по поводу заболевания. | O····O····O····O····O····O····O····O····O····O····O | | | | | | | 5. | | …подбодрила меня. | O····O····O····O····O····O····O····O····O····O····O | | | | | | | 6. | | …вселила в меня надежду на то, что я вновь смогу почувствовать себя лучше. | O····O····O····O····O····O····O····O····O····O····O | | | | | | | 7. | | …помогает мне участвовать в принятии решений относительно лечения. | O····O····O····O····O····O····O····O····O····O····O | | | | | | | 8. | | …показала мне, как я могу способство-вать успеху своего лечения. | O····O····O····O····O····O····O····O····O····O····O | | | | | | | 9. | | …придала мне мужества для принятию активных мер для улучшения моего состояния. | O····O····O····O····O····O····O····O····O····O····O | | | | | | |
| --- | --- | --- | --- | --- | --- | --- | --- | --- | --- | --- | --- | --- | --- | --- | --- | --- | --- | --- | --- | --- | --- | --- | --- | --- | --- | --- | --- | --- | --- | --- | --- | --- | --- | --- | --- | --- | --- | --- | --- | --- | --- | --- | --- | --- | --- | --- | --- | --- | --- | --- | --- | --- | --- | --- | --- | --- | --- | --- | --- | --- | --- | --- | --- | --- | --- | --- | --- | --- | --- | --- | --- | --- | --- | --- | --- | --- | --- | --- | --- | --- | --- | --- | --- | --- | --- | --- | --- | --- | --- | --- | --- | --- | --- | --- | --- | --- | --- | --- | --- | --- | --- | --- | --- | --- | --- | --- | --- | --- | --- | --- | --- |
