# Supplementary material for: Usefulness scale for patient information material (USE) - development and psychometric properties
Source: BMC Med Inform Decis Mak. 2015 Apr 19;15:34. doi: 10.1186/s12911-015-0153-7 (PMC4456699; doi:10.1186/s12911-015-0153-7)
Supplement: Additional file 7: — Usefulness scale for patient information material (USE) – Turkish. [file 12911_2015_153_MOESM7_ESM.doc]

**Additional File 7: Usefulness scale for patient information material (USE) – Turkish**

| Lütfen aşağıdaki tüm ifadeleri ne derece onayladığınızı belirterek değerlendirme yapınız. Bir ifadeye kesinlikle katılmıyorsanız, en solda bulunan daireyi işaretleyiniz. Bir ifadeye kesinlikle katılıyorsanız, en sağda bulunan daireyi işaretleyiniz. Bunların ortasında yer alan daireler onayınızın derecesini belirtebilme olanağını sağlar.  Yanlış işaretlediğinizde, bu işaretin üstünü çizebilir ve tekrar işaretleyebilirsiniz. Lütfen her ifadeye tek bir çapraz işareti koyunuz.  *Bunun nasıl yapılabileceğine ilişkin bir örnek:* Broşür, hastalığın tedavisini anlayabilmenizde size hiç yardımcı olmadıysa, aşağıdaki gibi işaretleyiniz:   | **Broşür…** | |  | | | | --- | --- | --- | --- | --- | |  | *kesinlikle*  *doğru değil*  X  *tarafsız*  X  *kesinlikle*  *doğru* | |  |  | | …tedaviyi anlamamda bana yardımcı oldu. | O····O····O····O····O····O····O····O····O····O····O | | | |   Lütfen her soruyu, mümkün olduğu kadar kendi şahsi görüşünüzü yansıtacak, açık ve dürüst bir  şekilde yanıtlayınız.  **Lütfen aşağıdaki ifadeleri değerlendiriniz:**   |  | **Broşür…** | | |  | | |  | | | --- | --- | --- | --- | --- | --- | --- | --- | --- | |  | *kesinlikle*  *doğru değil* | *tarafsız* | *kesinlikle*  *doğru* | |  | | 1. | | …benim için gerekli bilgileri içeriyor. | O····O····O····O····O····O····O····O····O····O····O | | | | | | | 2. | | …hastalığı anlamama yardımcı oldu. | O····O····O····O····O····O····O····O····O····O····O | | | | | | | 3. | | …hastalığın tedavi seçeneklerini anlamama yardımcı oldu. | O····O····O····O····O····O····O····O····O····O····O | | | | | | | 4. | | …hastalığımdan duyduğum endişeyi azalttı. | O····O····O····O····O····O····O····O····O····O····O | | | | | | | 5. | | …bana cesaret verdi. | O····O····O····O····O····O····O····O····O····O····O | | | | | | | 6. | | …kendimi tekrar daha iyi hissedebileceğime dair umut verdi. | O····O····O····O····O····O····O····O····O····O····O | | | | | | | 7. | | …tedavi hakkındaki kararlara katılmamda bana yardımcı oluyor. | O····O····O····O····O····O····O····O····O····O····O | | | | | | | 8. | | …bana tedavinin başarı ile gerçekleşmesinde benim nasıl katkıda bulunabileceğimi gösterdi. | O····O····O····O····O····O····O····O····O····O····O | | | | | | | 9. | | …durumumun düzelmesi için aktif olmam. | O····O····O····O····O····O····O····O····O····O····O | | | | | | |
| --- | --- | --- | --- | --- | --- | --- | --- | --- | --- | --- | --- | --- | --- | --- | --- | --- | --- | --- | --- | --- | --- | --- | --- | --- | --- | --- | --- | --- | --- | --- | --- | --- | --- | --- | --- | --- | --- | --- | --- | --- | --- | --- | --- | --- | --- | --- | --- | --- | --- | --- | --- | --- | --- | --- | --- | --- | --- | --- | --- | --- | --- | --- | --- | --- | --- | --- | --- | --- | --- | --- | --- | --- | --- | --- | --- | --- | --- | --- | --- | --- | --- | --- | --- | --- | --- | --- | --- | --- | --- | --- | --- | --- | --- | --- | --- | --- | --- | --- | --- | --- | --- | --- | --- | --- | --- | --- | --- | --- | --- | --- | --- |
